# Functional Antibody Response Against V1V2 and V3 of HIV gp120 in the VAX003 and VAX004 Vaccine Trials

**DOI:** 10.1038/s41598-017-18863-0

**Published:** 2018-01-11

**Authors:** Preetha Balasubramanian, Constance Williams, Mariya B. Shapiro, Faruk Sinangil, Keith Higgins, Arthur Nádas, Maxim Totrov, Xiang-Peng Kong, Andrew J. Fiore-Gartland, Nancy L. Haigwood, Susan Zolla-Pazner, Catarina E. Hioe

**Affiliations:** 1 The Sackler Institute of Graduate Biomedical Sciences, NYU School of Medicine, New York, NY 10016 USA; 2 Department of Medicine, Division of Infectious Diseases, Icahn School of Medicine at Mount Sinai, New York, NY 10029 USA; 3 Department of Pathology, NYU School of Medicine, New York, NY 10016 USA; 4 Division of Pathobiology and Immunology, Oregon National Primate Research Center, Oregon Health & Science University, Beaverton, OR 97006 USA; 5 Department of Molecular Microbiology & Immunology, Oregon Health & Science University, Portland, OR 97239 USA; 6 Global Solutions for Infectious Diseases, South San Francisco, CA 94080 USA; 7 Department of Environment Medicine, NYU School of Medicine, New York, NY 10016 USA; 8 Molsoft LLC, La Jolla, CA 92037 USA; 9 Department of Biochemistry and Molecular Pharmacology, NYU School of Medicine, New York, NY 10016 USA; 10 Vaccine and Infectious Disease Division, Fred Hutchinson Cancer Research Center, Seattle, WA 98109 USA; 11 James J. Peters VA Medical Center, Bronx, NY 10468 USA

## Abstract

Immunization with HIV AIDSVAX gp120 vaccines in the phase III VAX003 and VAX004 trials did not confer protection. To understand the shortcomings in antibody (Ab) responses induced by these vaccines, we evaluated the kinetics of Ab responses to the V1V2 and V3 regions of gp120 and the induction of Ab-mediated antiviral functions during the course of 7 vaccinations over a 30.5-month period. Plasma samples from VAX003 and VAX004 vaccinees and placebo recipients were measured for ELISA-binding Abs and for virus neutralization, Ab-dependent cellular phagocytosis (ADCP), and Ab-dependent cellular cytotoxicity (ADCC). Ab responses to V1V2 and V3 peaked after 3 to 4 immunizations and declined after 5 to 7 immunizations. The deteriorating responses were most evident against epitopes in the underside of the V1V2 β-barrel and in the V3 crown. Correspondingly, vaccinees demonstrated higher neutralization against SF162 pseudovirus sensitive to anti-V1V2 and anti-V3 Abs after 3 or 4 immunizations than after 7 immunizations. Higher levels of ADCP and ADCC were also observed at early or mid-time points as compared with the final time point. Hence, VAX003 and VAX004 vaccinees generated V1V2- and V3-binding Abs and functional Abs after 3 to 4 immunizations, but subsequent boosts did not maintain these responses.

## Introduction

Development of a prophylactic HIV vaccine has faced tremendous challenges. For most currently licensed vaccines, antibodies (Abs) or Ab-mediated functions induced by vaccines are immune correlates and surrogates of protection^1^. In the case of HIV, the virus envelope (Env), the only virus antigen present on the surface of virions and virus-infected cells, is the key target for Abs. The phase III VAX003 and VAX004 vaccine trials, designed to elicit protective Abs against HIV, utilized bivalent recombinant Env gp120 (rgp120) proteins AIDSVAX(B/E) and AIDSVAX(B/B), respectively. These trials demonstrated no efficacy in preventing HIV acquisition^2,3^. Nonetheless, subgroup analyses of the VAX004 trial indicated a trend toward greater efficacy among nonwhite and female participants^3^. Post hoc analysis of VAX004 further showed a trend toward lower HIV incidence among vaccinees with CD4-blocking and V2- and V3-binding Ab activity versus placebo recipients, and higher levels of these Abs were seen in nonwhite and female volunteers^4^.

Abs to V1V2 and V3 were identified as immune correlates for reduced risk of HIV acquisition in the RV144 vaccine trial^5,6^, which tested a prime-boost combination of the ALVAC-HIV(cCP1521) and AIDSVAX(B/E) vaccines and demonstrated vaccine efficacy of 31%^7^. Sieve analysis of breakthrough viruses from VAX003 vaccinees also showed a sieve effect at Env position 170 in the V2 region of gp120^8^; this differed from RV144 results, which showed a typical sieve effect at V2 position 169 and an atypical effect at V2 position 181^9^. Longitudinal analysis of VAX004 further showed that Ab responses to V2 appeared to peak after administration of 2 or 3 vaccine doses and did not improve with subsequent boosts^10^. Similarly, among VAX003 vaccinees, although Ab responses to V1V2 increased during the first 4 immunizations and reached higher levels than those of VAX004 vaccinees, levels declined with additional boosts^10^.

Studies of monoclonal antibodies (mAbs) have revealed distinct types of Ab epitopes on V1V2 and V3. Three types of epitopes were identified in the V1V2 β-barrel domain^11,12^. Conformation-dependent discontinuous V2i (integrin) epitopes (eg, epitopes of mAbs 830 A, 697, 2158) are located near the integrin α4β7-binding motif in the nonglycosylated underside of the V1V2 β-barrel^11,13^. The V2p (peptide) epitopes in the V2 C strand (eg, epitopes of CH58, CH59) are also devoid of glycan but can be represented by peptides^14^. The V2q (quaternary) epitopes, on the glycosylated upper side of V1V2, are dependent or preferentially presented on a V1V2 trimer (eg, epitopes of PG9, PG16, PGT145, PGDM1400)^15–19^. The V3 loop, particularly its crown, is also targeted by mAbs with distinct binding modes^20–22^. The ladle-type mAbs (eg, 447–52D) bind to the GPGR/Q arch of the V3 crown, and the cradle-type mAbs (eg, 2219, 2557) interact with the hydrophobic core in the circlet and band regions of the V3 crown without contacting the arch^23,24^. We previously demonstrated that, while chronic HIV infection leads to production of high levels of both V3 ladle- and cradle-type Abs, VAX003 and VAX004 vaccine recipients generated only cradle-type Abs, and their levels 2 weeks after the final boost were considerably low^22^. These findings raise the question of how Ab responses to these different epitopes on V1V2 and V3 were stimulated during the immunization period and whether stronger responses were induced at earlier time points but declined with additional boosts.

In this study, we examined the kinetics of Ab responses elicited against different types of V1V2 and V3 epitopes and the induction of Ab-mediated antiviral functions during the course of 7 vaccine doses over a 30.5-month period in VAX003 and VAX004 participants. Plasma samples from pre- and post-vaccination were measured for binding and functional Abs in ELISA, neutralization, antibody-dependent cellular phagocytosis (ADCP), and antibody-dependent cellular cytotoxicity (ADCC) assays. The data show that, although Ab responses to gp120 were stably maintained throughout the immunization period, Ab responses to V1V2 and V3 peaked after 2 to 4 vaccine doses and deteriorated with subsequent boosts. Importantly, virus neutralization, ADCP, and ADCC activities induced at early time points also waned after the final immunization.

## Methods

### Vaccines and Plasma Samples

VAX003 and VAX004 study designs were described^2,3^. The VAX003 protocol was approved by the ethics review committees of the Thailand Ministry of Public Health, Mahidol University, and the Bangkok Metropolitan Administration and by an institutional review board of the Centers for Disease Control and Prevention. The VAX004 study was conducted in accordance with the Declaration of Helsinki and local institutional review board requirements and with approval from appropriate regulatory authorities.

The VAX003 trial, conducted in injection drug users in Thailand, tested a bivalent AIDSVAX B/E vaccine that consisted of rgp120 proteins from HIV-1 strains MN (subtype B) and A244 (CRF01_AE)^2^. The VAX004 trial evaluated an AIDSVAX B/B vaccine made of 2 subtype B rgp120 proteins (MN and GNE8) in men who have sex with men and in women at high risk for heterosexual HIV transmission in North America and the Netherlands^3^. rgp120 (total 600 μg/dose) was produced in CHO cells and adsorbed onto alum. The placebo group received only alum. Vaccine and placebo recipients were injected intramuscularly at months 0, 1, 6, 12, 18, 24, and 30. Tested samples included plasma from 23 vaccine recipients and 8 placebo controls from each trial, collected before immunization and 2 weeks after each of the 7 injections. Participants were selected at random from the VAX003 and VAX004 studies, based on meeting the following criteria: 1) fully immunized, per protocol, 2) HIV-uninfected through the end of the study, and 3) plasma samples available at required time points. Sampling was stratified by sex to ensure a balance between females and males. All VAX003 samples were from nonwhite Asian volunteers (11 males and 12 females). For VAX004, samples were from 11 females (3 white, 7 black, and 1 Hispanic) and 12 males (10 white, 1 black, and 1 Hispanic).

### Recombinant Proteins and Peptides

rgp120 JRFL was purchased from Immune Technology. Case A2 V1V2 gp70 was provided by Dr. Abraham Pinter (PHRI)^25^. V1V2-tags of subtype C 1086 (V1V2_1086_-tags) were provided by Dr. Larry Hua-Xin Liao (Duke University). Except for V2 MN peptide (a gift of Dr. Nicos Karasavvas, USAMC-AFRIMS), all V3 and V2 peptides were synthesized by Biopeptide Co., Inc. (San Diego, CA). Biotinylated and nonbiotinylated peptides were used in ELISA and neutralization assays, respectively.

### ELISA Measurement of Antigen-specific IgG

ELISA was performed as described^22^, with updated modifications. Streptawell plates (Roche) were coated with biotinylated peptides (1 µg/mL, 1 h at 37 °C), followed by washing with PBS containing 0.05% Tween-20^**®**^ and blocking with RPMI containing 10% FCS (30 min at 37 °C). Diluted plasma samples were incubated for 1.5 h at 37 °C, and bound Abs were detected by alkaline phosphatase–conjugated anti-human IgG secondary Abs. After addition of substrate, optical density was read at 405 nm (OD_405_). The area under the titration curve (AUC) was calculated using Graphpad Prism version 7. Because the values of AUC and OD_405_ at 1:100 dilution correlated with each other (*P* = 0.0005; r = 0.597) (Figure S1), correlation of binding Abs with functional activities was done with either AUC or OD_405_.

### Virus-neutralization Assay

Virus neutralization was measured using TZM.bl target cells as described^22^. Each condition was tested in duplicate. Virus input corresponded to titrated stocks yielding 150,000 to 200,000 RLUs. Plasmids pNL4–3Δ *env* (from Dr. Nathaniel Landau) and SF162 *env* (from Drs. Leo Stamatatos and Cecilia Cheng-Mayer) used to produce pseudovirus in 293 T cells were obtained from the NIH AIDS Reagent Program. Neutralization inhibition was assessed using plasma pretreated with V3 peptide.

### ADCP Assay

Measurement of ADCP was done as described^26,27^, using THP-1 cells and fluorescent NeutrAvidin™ beads (1-µm diameter) precoated with a biotinylated, cyclic, full-length V3 peptide. Coated beads were incubated with serially diluted plasma, anti-V3 mAb (positive control), or irrelevant mAb (negative control) for 2 h at 37 °C; washed; and added to THP-1 cells. After overnight incubation, phagocytosis was measured by flow cytometry. ADCP scores were calculated as: (percentage of bead-positive cells × MFI of bead-positive cells)/10^6^, where MFI is mean fluorescence intensity.

### ADCC Assay

The Gran Toxilux^®^ ADCC assay was conducted as described^28^. Briefly, CEM.NKr-CCR5 target cells were coated with SF162 gp140, labeled with a cytoplasmic tracking dye TL4, and treated with diluted plasma. Target cells were then incubated with effector NK cells (rhesus CD16^+^ human NK cell line KHYG-1) at an effector/target ratio of 5:1 and treated with a fluorogenic granzyme B (GzB) substrate for 30 min at 37 °C. GzB delivered into target cells by effector cells was detected by flow cytometry. ADCC activity was calculated based on percentage of GzB^+^ target cells above background.

### Statistical Analysis

Comparison of Ab strength against gp120, V1V2, and V3 at peak vs final time points was done using the paired 2-tailed *t* test. Ab strength in male vs female volunteers at peak and final time points was compared using the unpaired 2-tailed *t* test. Neutralization and ADCC activities detected at different time points were compared using the ratio *t* test and paired 2-tailed *t* test, respectively. Correlation analyses were all performed with the nonparametric Spearman test.

### Data Availability

All data generated or analyzed during this study are included in this published article (and its Supplementary Information files).

## Results

### Plasma Ab responses to V1V2 and V3 peaked after 3 to 4 doses of immunization

**Figure 1:**
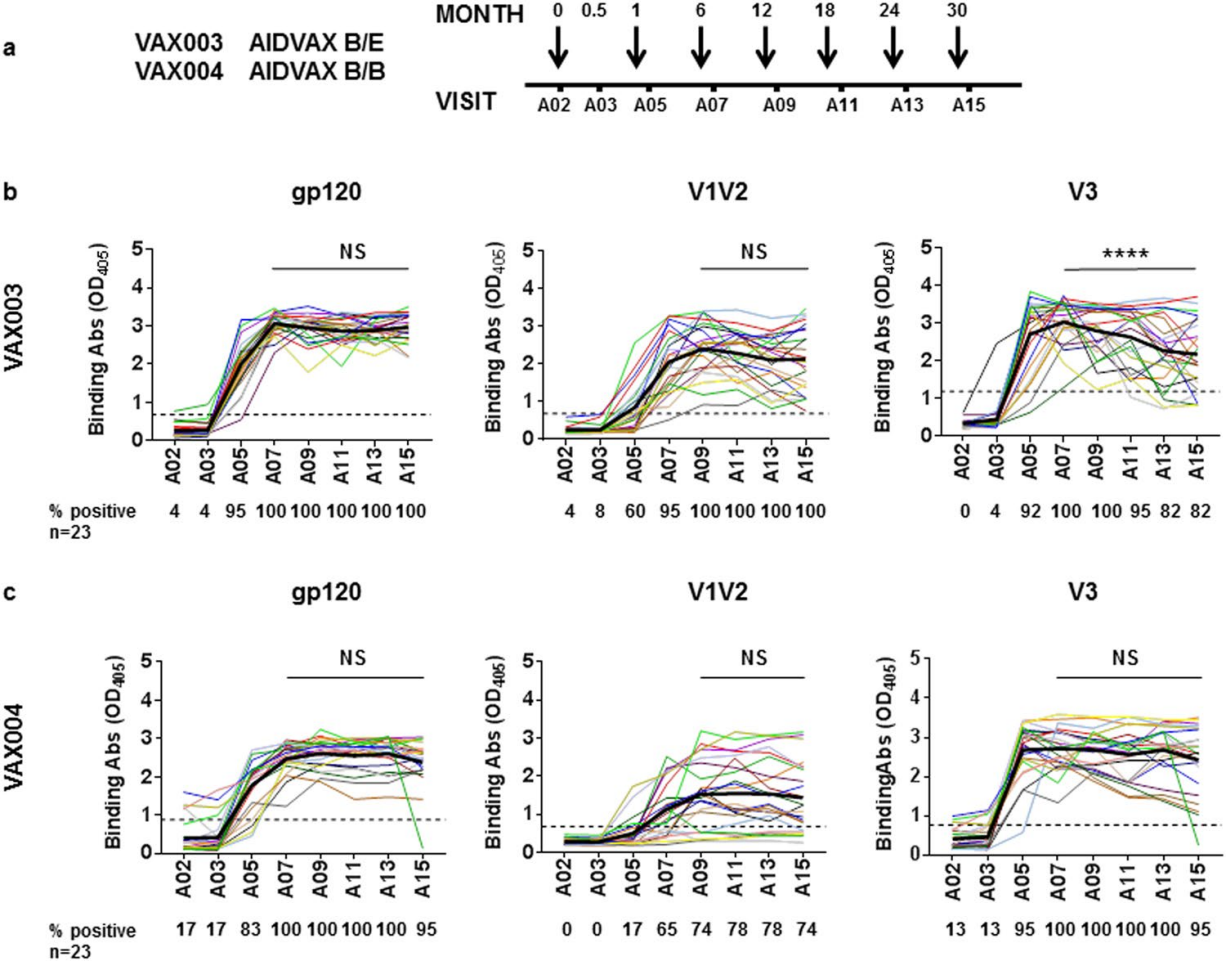
Plasma IgG responses to gp120, V1V2, and V3 in vaccine recipients in the course of 7 immunization doses. Plasma samples from 23 VAX003 (AIDSVAX^®^ B/E) and 23 VAX004 (AIDSVAX^®^ B/B) vaccine recipients were tested at 1:100 dilution for ELISA IgG reactivity against gp120 protein (B.JRFL), V1V2-gp70 protein (B.Case A2), and cyclic full-length 35-mer V3 peptide (B.MN). Samples were tested in duplicate against each antigen in 1 or 2 independent experiments; averages from all experiments are shown. (**a**) Plasma samples collected before immunization (A02) and 2 weeks after each of 7 vaccine doses (A03, A05, A07, A09, A11, A13, A15) were tested. (**b**) Longitudinal analysis assessed gp120-, V1V2-, and V3-binding Ab strengths in VAX003 vaccinees. **(c**) Longitudinal analysis of gp120-, V1V2-, and V3- binding Ab strengths was also performed for VAX004 vaccinees. Comparison of Ab strengths between A15 (final) and A07 or A09 (peak) was done by *t* test (paired, 2-tailed). Average Ab levels are highlighted by bold black lines. Percentages of responders (% positive) are shown below each graph. Cutoff values (dotted lines) were based on mean and 3 standard deviations of placebo recipients. OD_405_, optical density at 405 nm. ****P < 0.0001; NS: not significant (*P* ≥ 0.05).

Plasma samples from 23 participants each from the VAX003 and VAX004 trials were evaluated for ELISA reactivity against gp120 (B.JRFL), V1V2-gp70 (B.Case A2), and V3 (cyclic full-length 35-mer, B.MN). Plasma samples collected before immunization (A02) and 2 weeks after each of 7 vaccine doses (A03, A05, A07, A09, A11, A13, and A15) were evaluated. Plasma samples from 8 placebo recipients were also tested as negative controls. The data show that all volunteers in both trials generated anti-gp120 Abs after the third immunization (A07) and that levels of anti-gp120 Abs were maintained with subsequent boosts (A09-A15), except in 1 VAX004 participant (Fig. 1). Ab responses to V1V2-gp70 reached their peak after the fourth immunization (A09), but overall, Ab levels and response rates were higher in VAX003 vs VAX004 (Fig. 1). On average, anti-V1V2 Ab levels remained stable until the final immunization (A11-A15), although several VAX003 vaccinees had declining anti-V1V2 Ab responses following the fifth to seventh immunization doses. Anti-V3 Ab responses were also robustly induced and peaked after the third immunization, but for many vaccinees, anti-V3 Ab levels were not sustained with subsequent boosts. Among VAX003 participants, V3-binding Ab levels after the final dose (A15) were lower than those after the third and fourth immunizations (A07 and A09), and the percentage of responders declined to 82% from 100%. Many VAX004 participants also had declining anti-V3 Ab responses, but the decrease did not reach statistical significance. These results demonstrate that, while total Ab responses to gp120 were stable in all vaccinees during the entire immunization period of the VAX003 and VAX004 trials, Ab responses to V1V2 and V3 decreased in some vaccine recipients after 5 or more vaccine doses.

### Ab responses against V2i and V2p epitopes peaked after the third to fourth immunization and declined with subsequent boosts

**Figure 2:**
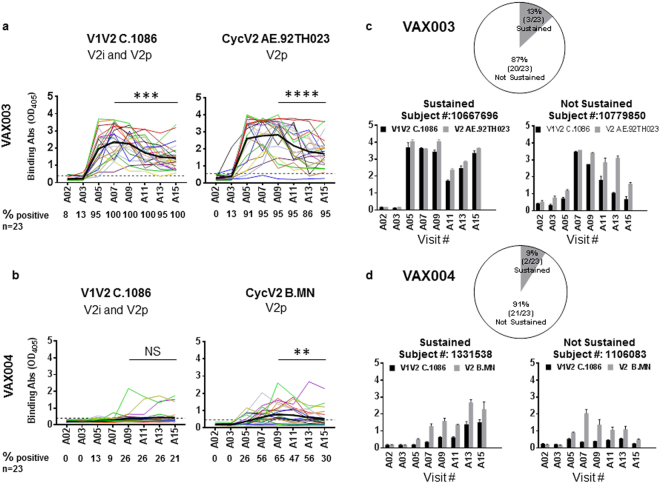
Anti-V1V2 plasma IgG responses in vaccine recipients over 7 immunization doses. Plasma samples from 23 VAX003 (AIDSVAX^®^ B/E) and 23 VAX004 (AIDSVAX^®^ B/B) trial participants were tested at 1:100 dilution for ELISA IgG reactivity against V1V2-tags (C.1086) expressing V2i and V2p epitopes and cyclic V2 peptides (AE.92TH023 or B.MN) bearing V2p epitopes only. Averages from 2 to 4 replicates tested in 1 or 2 independent experiments are shown. Plasma collection times are described in Fig. 1. (**a**) Longitudinal analysis compared induction of Ab responses to V1V2-tags (C.1086) and cyclic V2 peptide (AE.92TH023) in VAX003 vaccinees. **(b**) Similar analysis was performed on VAX004 samples to evaluate the kinetics of Ab responses to V1V2-tags (C.1086) and cyclic V2 peptide (B.MN). OD_405_, optical density at 405 nm. Percentages of responders (% positive) are shown below each graph. Cut-off values (black dotted lines) were calculated as mean and 3 standard deviations of placebo recipients. Mean antibody levels are highlighted by bold black lines. Comparison of Ab strengths at A15 and peak time points was done by *t* test (paired, 2-tailed). **P < 0.01, ***P < 0.001; ****P < 0.0001, NS: not significant. (**c**,**d**) Few VAX003 and VAX004 vaccinees had sustained Ab responses to V1V2-tags and V2 peptide. The majority of responses to one or both antigens decreased by > 30%. Data of 4 individual vaccine recipients (mean ± standard deviation) with sustained versus not sustained responses are shown.

Next we asked whether Abs induced in VAX003 and VAX004 participants target V2i (integrin) and V2p (peptide) epitopes, similar to mAbs isolated from HIV-infected or vaccinated humans. The reactivity of plasma Abs was tested against V1V2-tags protein (C.1086), recognizable by both V2i and V2p mAbs, and against cyclic V2 peptides reactive with only V2p mAbs^27^. The V1V2 C.1086 sequence had only 37.8% to 51.8% identity (47.8% to 66.2% similarity) with those of gp120 immunogens (B.MN, B.GNE8, E.A244) tested in the VAX003 and VAX004 trials, allowing detection of cross-reactive V2i-like Abs. Two V2 peptides (AE.92TH023 and B.MN) were used to detect the more strain specific V2p-like Abs. The data show that generally higher levels of Abs to V1V2 C.1086 and V2 peptides were detected among VAX003 (Fig. 2a) than among VAX004 (Fig. 2b) vaccinees. Only 6/23 (26%) VAX004 vaccinees produced detectable V1V2 C.1086-binding Abs (Fig. 2b). Ab responses to V2 peptide were stronger and observed in more VAX004 vaccinees at the peak A09 time point (65%), but they declined significantly at time points A11 to A15. The Abs were specific for V2 peptide of B.MN (Fig. 2b), without cross-reactivity with V2 AE.92TH023 (data not shown).

In contrast, all 23 VAX003 vaccine recipients had V1V2 C.1086-binding Ab responses, which peaked after the third immunization (A07), although Ab levels declined with subsequent boosts, and levels after the final boost (A15) were lower than those at or near peak (A07 and A09) (Fig. 2a). Similarly, Abs binding to cyclic V2 AE.92TH023 peptide were induced in most VAX003 vaccinees (95%), and responses decreased during the last 3 immunization doses (A11 to A15). Comparison of Ab strength at peak versus final time points demonstrated that in the majority of VAX003 vaccinees (87%), V1V2 Ab responses decreased by > 30% from peak levels (Fig. 2c). The same pattern was seen with VAX004, where Ab response to V2 peptide declined in 91% of vaccine recipients (Fig. 2d). These data indicate that V2i-like Abs cross-reactive with V1V2-tags were induced robustly in VAX003 but weakly in VAX004 vaccine recipients. Strain-specific V2 peptide-binding Abs were elicited in vaccine recipients in both trials, but the responses were stronger in VAX003 than in VAX004. Moreover, after reaching their peak with 3 to 4 vaccine doses, these responses deteriorated with additional boosts.

### Abs against the V3 crown were also unsustainable

We showed previously that the majority of VAX003 and VAX004 vaccinees had Ab responses to V3 cradle-type epitopes in the immunogenic crown of the V3 loop after the final boost, and that very few produced the V3-ladle-type Abs that bind to the GPGR/Q arch of the V3 crown^22^. To better characterize the kinetics by which these different types of Abs were induced in both sets of trial participants, we examined longitudinal plasma samples in ELISA for IgG levels to V3 crown, V3 cradle, and V3 ladle. Abs to V3 crown were detected using a linear 13-mer peptide, whereas V3 cradle- and ladle-type Abs were detected using cyclic peptide mimotopes that distinguish these 2 Ab types^20,22^.

**Figure 3:**
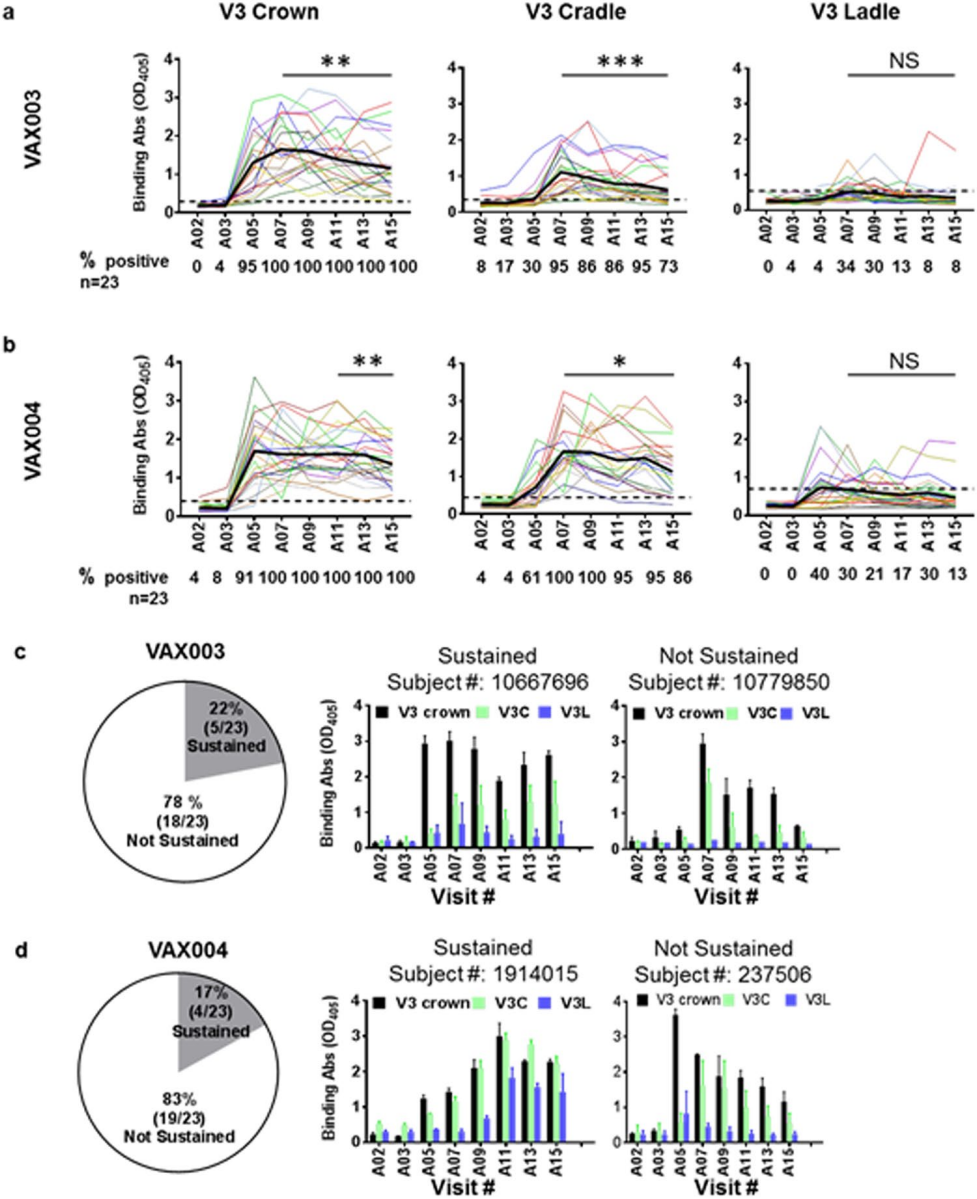
Anti-V3 crown plasma Ab responses in VAX003 and VAX004 vaccine recipients over 7 immunization doses. ELISA reactivity of plasma IgG from 23 VAX003 and 23 VAX004 vaccinees was assessed against linear V3 crown peptide, cyclic V3 cradle peptide, and cyclic V3 ladle peptide. All peptides were biotinylated. Plasma was tested at prebleed (A02) and 2 weeks after each AIDSVAX B/E vaccine dose (A03-A15). Averages from 4 replicates tested in 2 independent experiments are shown. **(a**,**b**) Kinetics of Ab responses induced to V3 crown, V3 cradle, and V3 ladle peptides in VAX003 (**a**) and VAX004 vaccinees (**b**). OD_405_, optical density at 405 nm. Percentages of responders (% positive) are shown below each graph. Cutoff values (black dotted lines) were calculated as mean and 3 standard deviations of placebo recipients. Mean antibody levels are highlighted in bold black lines. *P < 0.05, **P < 0.01, ***P < 0.001; NS: not significant by *t* test (paired, 2-tailed). **(c**,**d**) In the vast majority of VAX003 (**c**) and VAX004 (**d**) vaccine recipients, Ab responses against V3 crown and V3 cradle arose after 2 to 3 doses and declined by > 30% with subsequent doses. Data from representative individuals (mean ± standard deviation) with sustained versus not sustained responses are shown.

The data in Fig. 3 show that Abs to V3 crown were detected in 100% of VAX003 and VAX004 vaccinees after 3 doses (A07), and all remained positive until the final dose (A15). Average levels of V3 crown-binding Abs peaked at A07 but declined with subsequent boosts. For VAX003 vaccinees, V3 crown-binding Ab levels at A15 were lower than those at or near the peak (A07 and A09; *P* = 0.020 and 0.001, respectively), whereas among VAX004 vaccinees, the decline was evident at later time points, from A11 and A13 to A15 (*P* = 0.041 and 0.009, respectively) (Fig. 3a,b).

The induction of V3 cradle- and ladle-type Abs was more transient. Although V3 cradle-type Abs were induced in 95% of VAX003 and 100% of VAX004 vaccinees after 3 doses (A07), the percentage of responders decreased to 73% and 86%, respectively, after the final dose (A15) (Fig. 3a,b). Comparison of binding strengths for individual vaccinees between peak vs final time points also showed that binding strength declined in both VAX004 and VAX003 vaccinees, and the decrease was more precipitous for participants in VAX003 than VAX004. V3 cradle-type Ab strength was also lower for VAX003 participants. Consistent with our previous findings^22^, V3 ladle-type Abs were produced by fewer VAX003 and VAX004 vaccinees, and their binding strength was much lower compared with that of V3 cradle-type Abs. Nonetheless, at peak A07 and A05 time points, 34% and 40% of vaccinees were positive for V3 ladle-type Abs. There were negligible boosting effects with subsequent vaccine doses, and for many vaccinees in both trials, V3 cradle- and ladle-type Abs were detected transiently, at 1 or 2 time points only.

When Ab binding strengths against V3 crown, V3 cradle, and V3 ladle of individual vaccinees were compared, 78% of VAX003 and 83% of VAX004 vaccine recipients had Ab responses that dropped by > 30% at the final time point (A15) as compared with the peak (Fig. 3c,d), similar to the V1V2 Ab data (Fig. 2). Also, only 1 VAX003 and 3 VAX004 vaccinees displayed sustained Ab responses to both V1V2 and V3. Table 1 summarizes significant changes observed in Ab binding strength from peak to final time points in VAX003 and VAX004 volunteers. Taken together, the data demonstrate that Ab responses against immunogenic sites in V1V2 and V3 were induced initially in most VAX003 and VAX004 vaccinees, but vaccination after 4 doses did not boost the responses and may have exerted suppressive effects.

**Table 1:** Comparison of binding and functional Ab responses induced in VAX003 and VAX004 vaccine recipients at peak vs final time points^a^.

	VAX003	VAX004
Binding Abs^b^		
gp120	NS	NS
V1V2-gp70 (B.Case A2)	NS	NS
V1V2-tags (C.1086)	***	NS
CycV2 (AE.92TH023 or B.MN)	****	**
V3 full length (B.MN)	****	NS
V3 crown	**	**
V3 cradle	***	*
V3 ladle	NS	NS
	**VAX003 & VAX004**	
**Functional Abs** ^**c**^		
Neutralization (B.SF162)	**	
V3 Ab Neutralization	**	
V3 ADCP	ND	
gp120 ADCC	**	

^a^Responses at peak and final time points were compared by *t* test (paired, 2-tailed).

^b^ELISA data from Figs 1, 2 and 3.

^c^Data from Figs 5, 6, 7 and 8.

NS: not significant (*P* ≥ 0.05), *P < 0.05, **P < 0.01, ***P < 0.001, ****P < 0.0001.

ND: statistical analysis was not done.

### Ab responses against V2 and V3 were more sustained in female vs male participants in the VAX004 but not the VAX003 trial

Because subgroup analyses of the VAX004 trial indicated vaccine efficacy among female and nonwhite volunteers, we compared Ab responses to V1V2 and V3 in female vs male vaccine recipients. Seventy-three percent (8/11) of female volunteers from the VAX004 study were nonwhite, whereas 83% (10/12) of male volunteers were white; in the nonwhite female and white male subgroups, vaccine efficacy was 74% (*P* = 0.10) and −6% (*P* = 0.61), respectively^3^.

**Figure 4:**
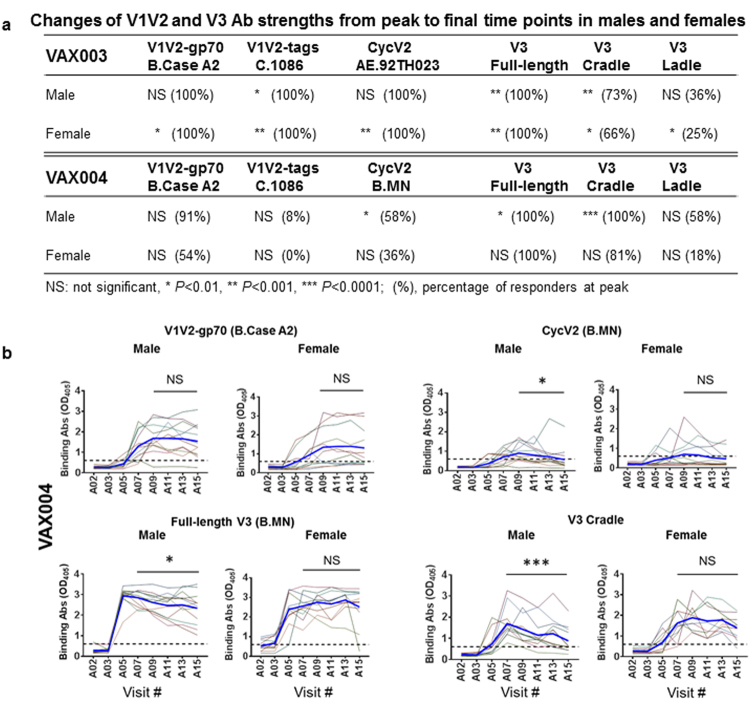
Comparison of Ab responses to V1V2 and V3 in males and females at peak vs final time points. Plasma samples from 11 male and 12 female VAX003 vaccinees and 12 male and 11 female VAX004 vaccinees were tested at 1:100 dilution for ELISA IgG reactivity against V1V2-gp70 (B.Case A2), V1V2-tags (C.1086), Cyc V2 peptides (AE.92TH023 or B.MN), full-length V3 (B.MN), V3 cradle, and V3 ladle. Samples from A02 visit (prebleed) and 2 weeks after each vaccination (visits A03-A15) were tested. Each sample was tested in 2 to 4 replicates in 1 or 2 independent experiments. (**a**) Changes of Ab levels from peak (A07 or A09) vs final (A15) time points were evaluated for male vs female vaccinees by paired, 2-tailed *t* test. Percentages of responders at peak were also calculated. (**b**) Representative graphs show declining Ab responses to V1V2 and V3 in male but not in female participants of the VAX004 study. Average Ab levels are highlighted by bold blue lines. Cutoff values (dotted lines) were based on mean and 3 standard deviations of placebo recipients. Comparison of Ab strength between A15 (final) and A07 or A09 (peak) was done by *t* test (paired, 2 tailed). *P < 0.05, **P < 0.01, ***P < 0.001, NS: not significant (*P* ≥ 0.05). OD_405_, optical density at 405 nm.

The data in Fig. 4a,b demonstrate that Ab responses to V1V2 (V1V2-gp70; B.Case A2) in female and male VAX004 participants were maintained similarly after reaching their peak at time point A09. Anti-V1V2 Ab strength at peak and final time points also was not different between sexes. Ab responses to V1V2 C.1086 and CycV2 B.MN were weak in both males and females, but responses to CycV2 B.MN declined in males (*P* = 0.015 between final A15 vs peak A09) as compared with females at the same time points (*P* > 0.05). A more pronounced difference was seen with anti-V3 Abs, in which declining Ab responses to full-length V3 and V3 cradle were seen from A07 to A15 time points in males but not in females. V3 ladle-type Ab responses, on the other hand, were weak and transient in both sexes. The distinct pattern observed in anti-V2 and V3 Ab responses between male vs female VAX004 vaccinees was not seen in the VAX003 study, in which female vaccinees had Ab responses to all 6 tested antigens that declined from peak (A07 or A09) to A15 time points, whereas male vaccinees had diminishing Ab responses only to 3 antigens: V1V2 C.1086, full-length V3, and V3 cradle (Fig. 4a). These data demonstrate that in the VAX004 study, female vaccinees had more-sustained Ab responses to V2 and V3 than did their male counterparts, while in the VAX003 study, female participants displayed less-sustained responses.

### Neutralizing Ab responses induced in many vaccinees declined after final immunization

**Figure 5:**
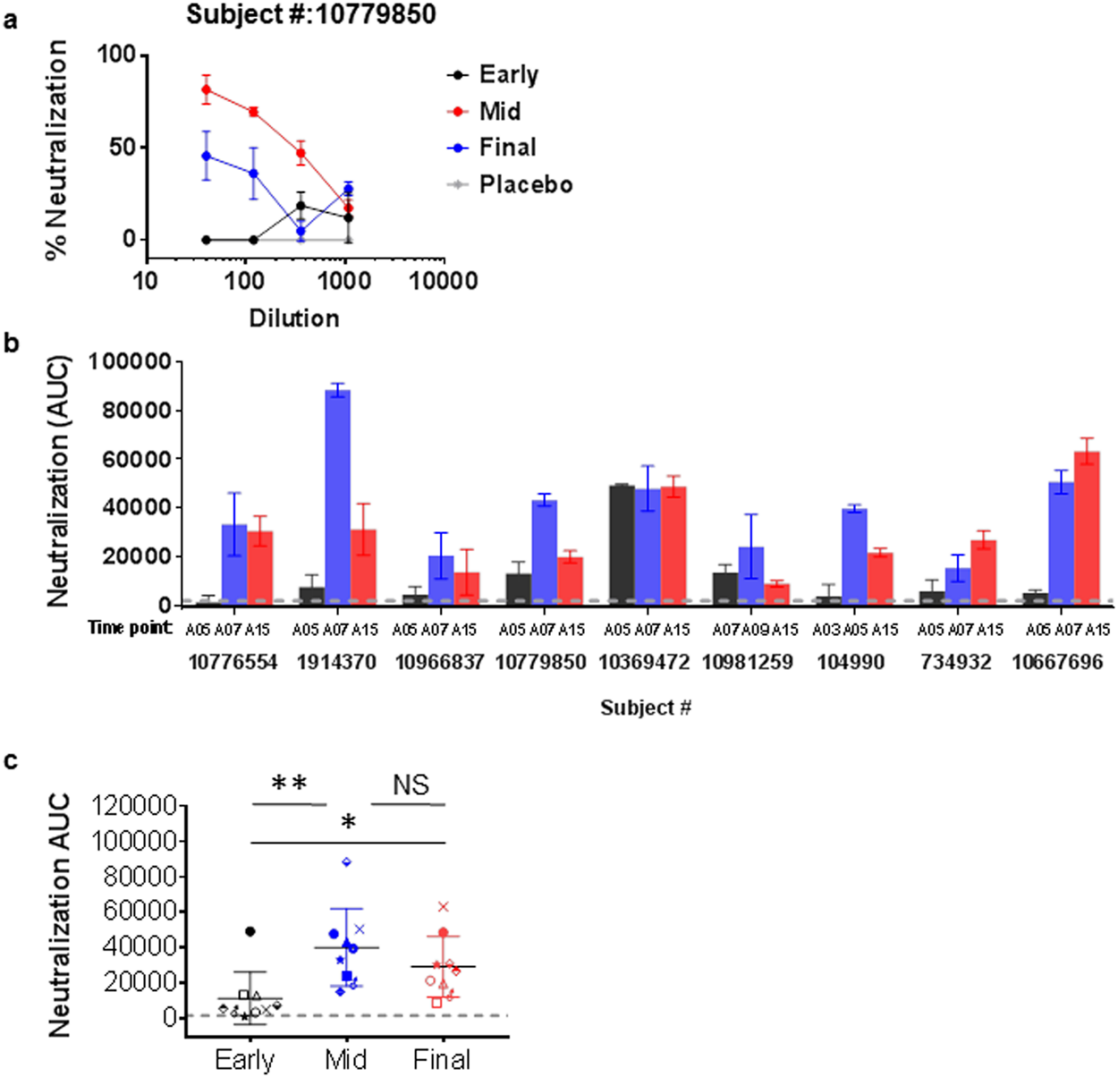
Changes of virus-neutralizing activity detected in plasma of vaccinees during immunization. Plasma samples from VAX003 and VAX004 vaccinees (n = 9 total) were assessed for neutralization against SF162 pseudovirus. For each vaccinee, plasma from 3 time points was selected based on changes in ELISA anti-V3 Ab strength before peak, at peak, and at final A15 time point. Samples were tested in 2 to 4 replicates in 1 or 2 independent experiments. **(a**) Neutralization of titrated plasma from early, mid-, and last time points from 1 vaccinee. Averages and standard deviation are shown. Plasma was diluted 3-fold from 1:40 to 1:1080, and area under the curve (AUC) was calculated from the titration curve using Graphpad Prism version 7. **(b**,**c**) Neutralization activities (AUC ± standard error) at early, mid-, and final time points were compared. Most vaccinees displayed neutralization that peaked at mid-time points, coinciding with the peak of anti-V3 Ab responses, and declined after the final boost. Statistical comparison was performed using the ratio paired 2-tailed *t* test. *P < 0.05, **P < 0.01; NS: not significant.

Because V3-specific Abs have neutralizing activity against sensitive tier 1 HIV isolates, we asked whether virus-neutralizing Ab responses were also induced transiently in VAX003 and VAX004 trial participants and deteriorated by the end of the immunization period. Because of sample amount limitation, we assessed neutralizing activities of plasma samples from 9 vaccinees collected at 3 time points: early time points before the peak of ELISA binding strength, peak time points of V3 crown- and cradle-binding strengths (A07 except for 2 vaccinees with peak time points of A09 and A05), and A15 time point at 2 weeks after the final boost (Fig. 5).

Of the 9 vaccinees tested, 6 were VAX003 and 3 were VAX004 participants, but their neutralization profiles were indistinguishable. Similar to participant #10779850 shown in Fig. 5a, most vaccinees had no neutralization activity against tier 1 SF162 virus at the early time point, but neutralization arose at the mid-time point and declined at the final time point (Fig. 5b,c). Few vaccinees (e.g. #10369472, #734932 and #10667696) displayed stable or increasing neutralization (Fig. 5b). Neutralization area under the curve (AUC) correlated with ELISA Ab strength to gp120 (r = 0.518; *P* = 0.005 by nonparametric Spearman correlation test) and V1V2 Case A2-gp70 (r = 0.513; *P* = 0.006) but not with V3-binding Ab levels (r = 0.277; *P* = 0.161), indicating that Abs targeting sites other than V3 contribute to virus neutralization measured.

**Figure 6:**
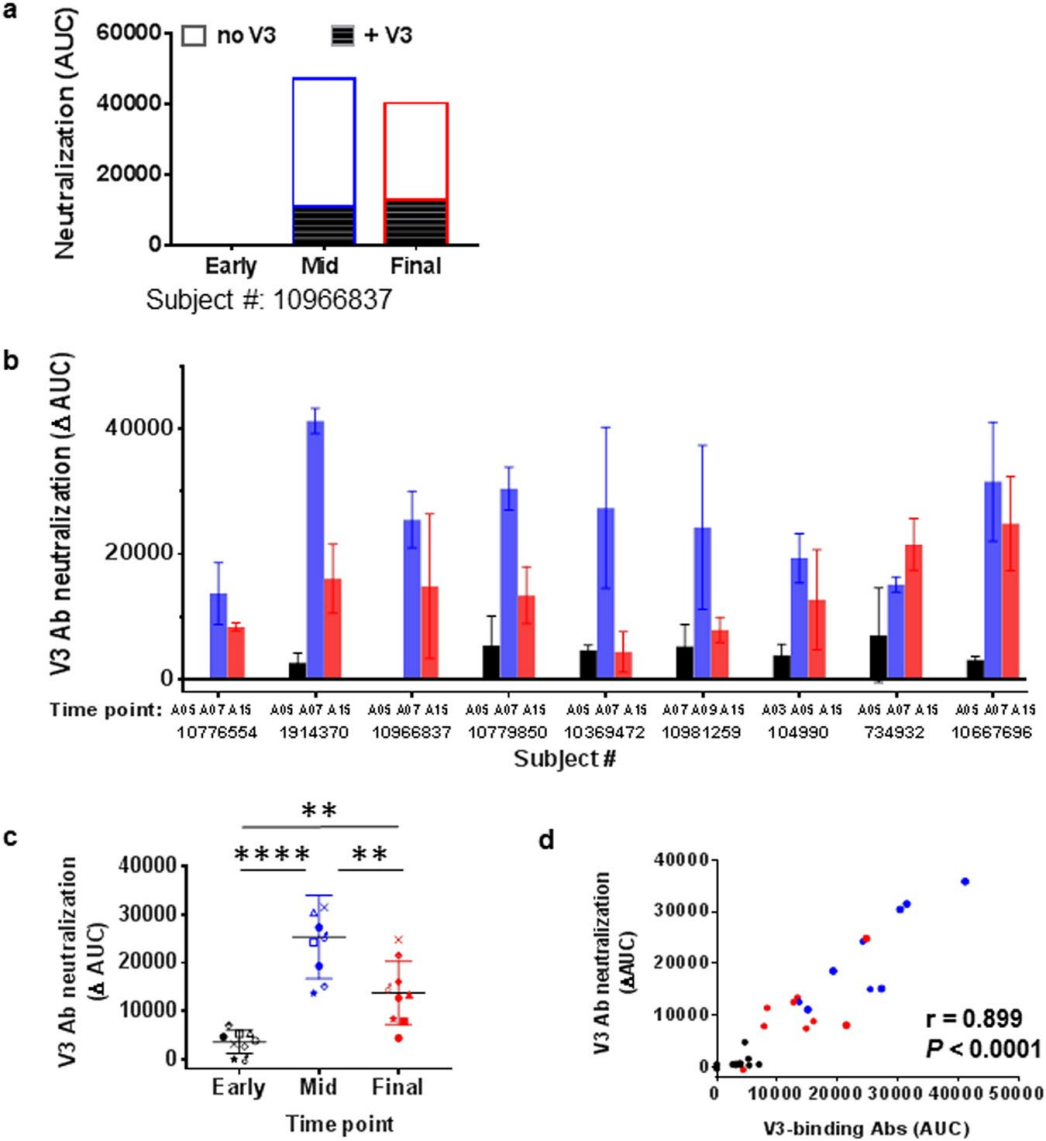
Changes of anti-V3 Ab-mediated neutralization in vaccine recipients in the course of immunization. Samples from 6 VAX003 and 3 VAX004 participants were tested in duplicates. **(a**) Neutralization mediated by anti-V3 Abs was assessed by measuring neutralization activity of plasma treated with or without V3 peptide (20 µg/mL, B.MN sequence, cyclic and nonbiotinylated) against SF162. Areas under the curve (AUC) of untreated and V3-peptide-treated plasma from early, mid-, and final time points are shown for 1 vaccinee. ∆AUC (open bar) was calculated by subtracting AUC of untreated plasma (top bar) from that of treated plasma (solid bar). **(b**,**c**) Neutralization by V3 Abs was undetectable at early time points (black), arose at mid-time points (blue), and declined after the final boost (red). ∆AUC ± standard errors are shown. **P < 0.01, ****P < 0.0001by ratio paired 2-tailed *t* test. **(d**) ∆AUC values correlated with ELISA V3-binding Ab levels detected in corresponding plasma samples. Correlation analysis was performed using the nonparametric Spearman test.

To determine virus neutralization contributed specifically by anti-V3 Abs, neutralization assays were performed with plasma pretreated with blocking V3 peptide. V3-mediated neutralization is represented as ∆AUC, calculated by subtracting neutralization by plasma treated without peptide from neutralization in the presence of V3 peptide (Fig. 6a). For most vaccinees, no or little ∆AUC was detected at the early time point; ∆AUC was greatest at the mid-time point and coincided with the peak of V3-binding Ab strength; and ∆AUC was reduced at the final time point (Fig. 6b,c). V3 Ab-mediated neutralization measured by ∆AUC correlated with ELISA V3-binding Ab levels (r = 0.899; *P* < 0.0001 by Spearman test) (Fig. 6d). These results show that virus-neutralizing, V3-specific Ab responses were induced in VAX003 and VAX004 vaccinees at mid-points of the immunization period but dropped after the final boost.

### ADCP and ADCC induced in vaccinees also declined after final immunization

**Figure 7:**
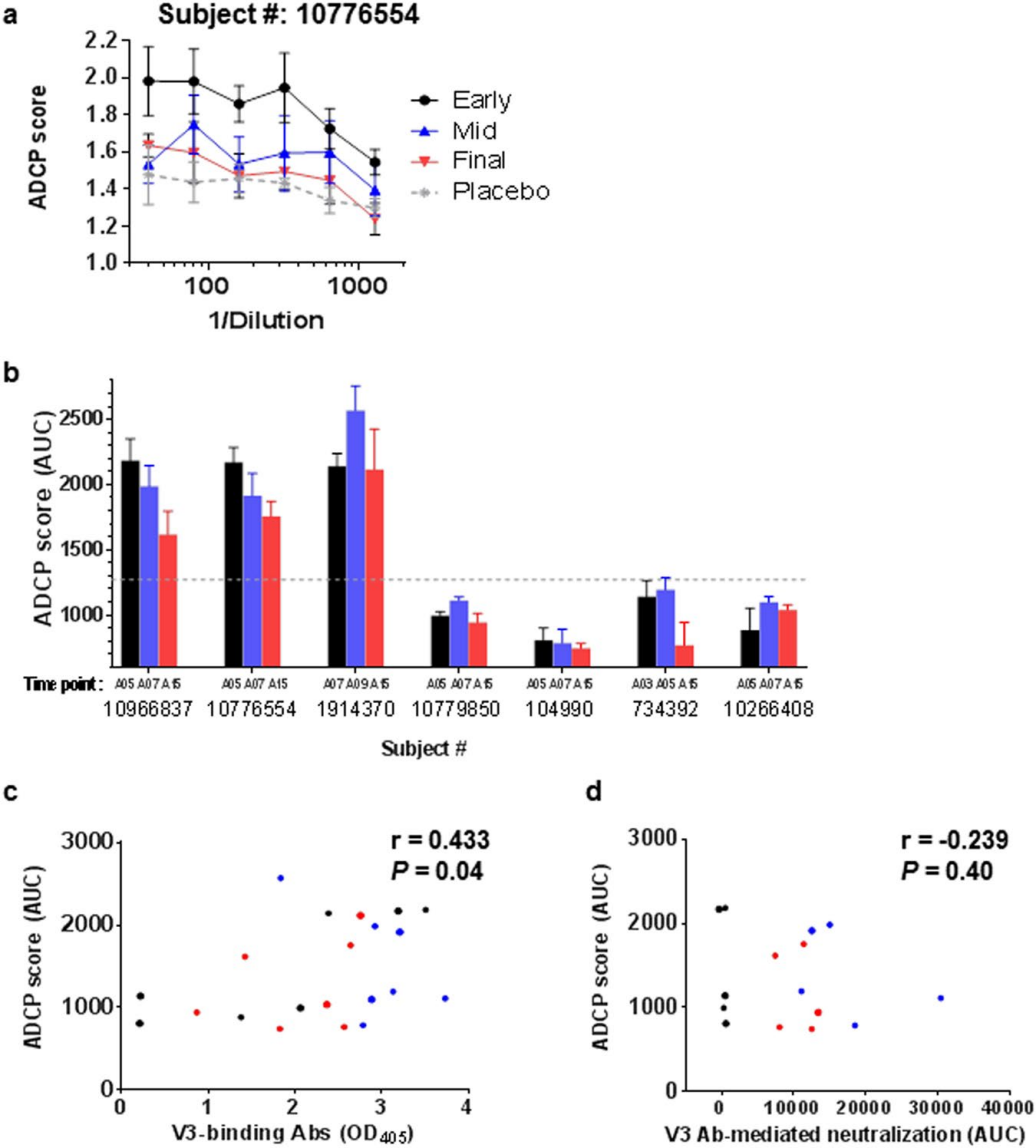
Induction of anti-V3 Abs with phagocytic activity in vaccine recipients. The phagocytic activity of anti-V3 Abs in plasma of 7 vaccinees (4 VAX003 and 3 VAX004) was assessed using fluorophore Neutravidin™ beads coated with a biotinylated, cyclic, full-length V3 peptide (4 µg V3 peptide mixed with 2 µg scrambled peptide for 10 µL of beads/96 wells) for 2 h at 37 °C, washed, and resuspended in PBS with 0.1% BSA. Beads (9 × 10^5^/10 µL/well) were incubated with serially diluted plasma, anti-V3 mAb, or irrelevant mAb for 2 h at 37 °C, washed, and added to THP-1 cells (2.5 × 10^4^/well; ATCC^®^). After overnight incubation, phagocytosis was measured by flow cytometry. ADCP score was calculated by multiplying the percentage of bead-bearing cells with geometric mean intensity of the cells and subtracting background score. **(a**) Antibody-dependent cellular phagocytosis (ADCP) activities of plasma from 1 vaccinee were diluted 2-fold 6 times from 1:40. Plasma samples from early, mid-, and final time points were compared with plasma from a placebo recipient. Averages and standard error from 4 replicates tested in 2 independent experiments are shown. **(b**) ADCP activities were measured in plasma from 7 vaccinees collected at early, mid-, and final time points and shown as area under the curve (AUC ± standard error) of ADCP scores. Cutoff value (dotted lines) was determined based on mean plus 3 standard deviations of placebo ADCP scores. **(c**) Correlation between ADCP score and ELISA V3-binding Ab levels was analyzed by the Spearman test. (**d**) V3-specific ADCP and V3 Ab-mediated neutralization activities were correlated by the Spearman test.

Next we examined whether anti-V3 Abs induced in VAX003 and VAX004 vaccinees can mediate ADCP and whether ADCP activities are maintained by administration of 7 vaccine doses. ADCP was performed using V3 peptide-coated fluorophore beads to measure specifically anti-V3 Ab-mediated ADCP activity. Of 7 vaccinees tested (4 from VAX003 and 3 from VAX004), only 2 VAX003 and 1 VAX004 vaccinee showed ADCP activity, which appeared earlier than virus neutralization: after the second or third dose (A05 or A07) (Fig. 7a,b). ADCP was retained at the final time point, A15, although scores at A15 tended to be lower than early or mid-point scores. ADCP scores correlated with levels of V3-binding IgG (Fig. 7c). However, these V3-specific ADCP scores did not correlate with V3 Ab-mediated neutralizing activities (Fig. 7d).

**Figure 8:**
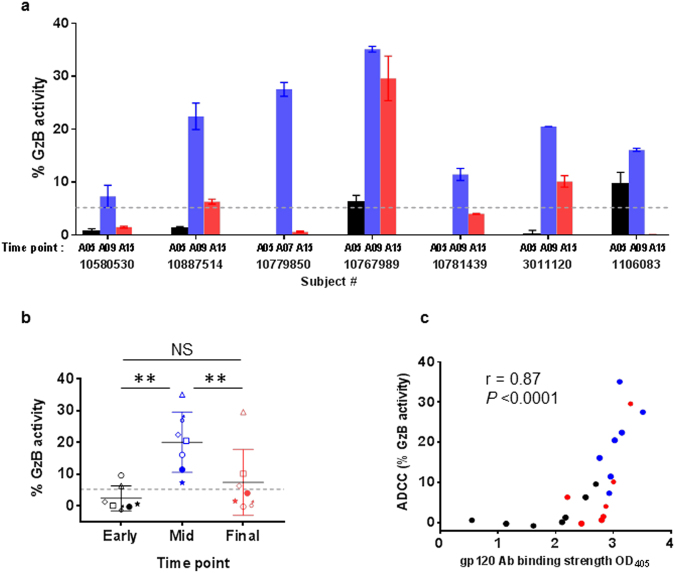
Induction of ADCC activity in vaccine recipients. Antibody-dependent cellular cytolysis (ADCC) activity was measured using CEM.NKr-CCR5 target cells coated with SF162 gp140 (5 µg/100,000 cells/sample), labeled with TL4 dye, and treated with diluted plasma (1:200) and with rhesus CD16^+^ human NK KHYG-1 effector cells at an effector/target ratio of 5:1. The mixtures were then suspended in a granzyme B (GzB) substrate for 30 min at 37 ^o^C. GzB delivered into target cells by effector cells was detected with the fluorogenic GzB substrate and visualized by flow cytometry. ADCC was calculated based on percentage of GzB^+^ target cells above negative control. Cut-off value (dotted line) was determined as the mean plus 3 standard deviations of % GzB^+^ cells in pooled prebleed plasma. **(a**) ADCC activities were measured in plasma of 5 VAX003 and 2 VAX004 vaccinees collected at early (A05, after 2 vaccine doses), mid- (A07 or A09, after 3 or 4 doses), and final (A15, after 7 doses) time points. Averages and standard deviation of duplicate wells from an experiment are shown. **(b**) ADCC activity was detected at mid-time points (A07 or A09) but declined at A15. **P > 0.01; by the paired 2-tailed *t* test. **(c**) ADCC activity strongly correlated with anti-gp120 Ab levels detected in ELISA. Correlation analysis was performed using the nonparametric Spearman test.

Finally, we examined ADCC activity in plasma from 7 vaccinees (5 from VAX003 and 2 from VAX004) using gp140-coated target cells, which detected ADCC mediated by total anti-gp120 Abs. ADCC activities were undetected or low after 2 vaccine doses (A05) but were detected in the plasma of all 7 vaccinees after 3 or 4 doses (A07 or A09) (Fig. 8a,b). However, like neutralization activity, ADCC levels decreased after 7 doses (A15) (*P* < 0.01 vs mid-time points, Fig. 8b). At A15, only 1 vaccinee still had high ADCC, and the remaining 6 had low or no ADCC. ADCC activity correlated with levels of Abs to gp120 (Fig. 8c), V1V2 (r = 0.638; *P* = 0.002), and V3 (r = 0.575; *P* = 0.006).

Taken together, these data demonstrate the induction of Abs against V1V2 and V3 that display antiviral functions in vaccine recipients of the VAX003 and VAX004 trials. These Abs arose early or at mid-time points in the immunization period, but the responses declined and were lost after the final boost.

## Discussion

This study highlights a significant impediment in the induction of Ab response by VAX003 and VAX004 AIDSVAX gp120 vaccines that was not appreciated previously. The data revealed that, although overall IgG Ab responses to gp120 were elicited early and maintained throughout the 2.5 years of the immunization period, responses to V1V2 and V3 were transiently elicited in the vast majority of vaccinees, most evidently against specific types of V1V2 and V3 epitopes detected with V1V2-tags, V2 peptide, V3 cradle, and V3 ladle antigens (Table 1). Of note, responses to these V1V2 and V3 epitopes declined after 5 to 7 vaccine doses among male VAX004 participants but not among female counterparts, a subgroup in whom a greater, albeit nonsignificant, vaccine efficacy was demonstrated^3^. In contrast, among VAX003 participants, both males and females showed decreasing responses to V1V2 and V3, and female responses tended to drop more (Fig. 4).

The decline of V1V2-specific IgG responses observed here is consistent with a recent study showing Ab responses to V1V2 Case A2 in VAX003 and VAX004 vaccinees peaked after the fourth immunization (A09) and did not increase with two additional boosts (A13)^10^. V1V2-binding Ab responses in vaccine recipients in the RV144 vaccine trial were also short-lived, and vaccine efficacy declined to 31% at 3.5 years from 60% at year 1^29^. When 2 additional boosts were administered to RV144 vaccinees 6 to 8 years later, Ab titers to gp120 and V1V2 attained after the second vaccine dose were lower than those induced by the first dose^30^. A recent study by Chung *et al*.^31^ evaluating gp120-specific IgG subtypes in the VAX003 and RV144 trials demonstrated that among VAX003 volunteers, total IgG titers increased and were sustained from mid- to final time points (A05, A07, and A15), consistent with data from our study. However, anti-gp120 IgG2 and IgG3 subtypes decreased, whereas IgG1 and IgG4 counterparts increased. IgG4 specific for V1V2 was also augmented after 7 vaccinations in the VAX003 study; this pattern was not seen in the RV144 trial^31^. In this study the declining Ab response to specific types of V1V2 epitopes (V2i and V2p) was observed in the context of total IgG. With regard to anti-V3 Ab responses, V3 cradle-type IgG responses were more robust than V3 ladle-type responses for VAX003 and VAX004 vaccinees, in agreement with our earlier cross-sectional study^22^. However, this longitudinal study revealed that most individuals had detectable responses only at 1 or 2 mid-time points. Overall, only 5/46 vaccinees had sustained IgG responses to the specific V1V2 and V3 epitopes throughout the immunization period.

The functional activities of Abs measured in virus neutralization, ADCP, and ADCC assays also decreased at the final time point, after being induced to higher levels at early to mid-time points (Table 1). These results demonstrate that the initial 3 to 4 immunizations with AIDVAX rgp120 proteins efficaciously primed and boosted Ab responses against diverse epitopes on V1V2 and V3 and that the induced plasma Abs had neutralizing and nonneutralizing antiviral potential, but additional injections of the same vaccines were not advantageous and, in fact, appeared to be detrimental for maintaining functional Ab responses.

A possible explanation for the weakening boosting capacity of rgp120 vaccines after 5 or more immunization doses is that at later time points, IgG Abs against other gp120 regions may have been elicited and supplanted the responses to V2i, V2p, V3 cradle, and V3 ladle. However, the specific regions targeted by Abs prevailing after 5 doses have yet to be defined. IgG to the C1 and C5 regions of gp120 was found to be among the dominating Ab responses in VAX003 and VAX004 vaccinees 2 weeks after the fourth immunization, but responses to these regions following subsequent boosts were not evaluated^6^. High levels of Ab responses to V1V2 and V3, which are the most immunogenic regions on gp120, were induced after 2 to 3 vaccine doses in all 46 VAX003 and VAX004 participants evaluated in this study. V1V2- and V3-specific Abs are also readily generated by various Env vaccines and during HIV infection^32–41^. However, on soluble gp120 monomers, V1V2 and V3 loops do not adopt fixed structural conformations^11,14,16,17,42–44^. As these flexible loops are constantly flickering, we propose that only a small fraction of memory B cells that are primed during the first 2 to 3 immunizations may be able to engage V1V2 and V3 configurations specifically recognizable by their B-cell receptors. In support of this idea, more durable Ab responses to V1V2 and V3 that lasted for ≥ 1 year were observed in animals immunized with scaffold immunogens that present V1V2 and V3 in more constrained configurations than do the native gp120 proteins^27,37,45^. Additionally or alternatively, anti-V1V2 and anti-V3 Abs that are induced early may form stable immune complexes with rgp120 introduced in later boosts, preventing recognition and stimulation of naive and memory B cells specific for these epitopes. Nonetheless, with 6-month intervals between immunizations, levels of V2- and V3-specific Abs in the plasma at the time of immunization were low, having already declined to reach the trough^4^.

Functional correlates for protection against HIV remain unclear. Abs against V1V2 and V3 induced by rgp120 proteins in the VAX003 and VAX004 trials and by other Env-vaccine candidates tested thus far neutralize mainly tier 1 viruses and few tier 2 viruses. V1V2 and V3 Abs also mediate Fc-dependent functions such as ADCP and ADCC^46,47^. ADCC activity of plasma IgG, including V1V2-specific IgG3, showed inverse correlation with HIV infection risk in the RV144 vaccinees with low levels of Env-specific IgA^5,48^. An early study by Forthal *et al*. further showed that Ab-dependent cellular viral inhibition of Abs induced in the VAX004 trial correlated with reduced rates of infection^49^. Our study demonstrates for the first time that neutralization against tier 1 SF162 was induced to the highest magnitude after 2 to 4 immunization doses in each of VAX003 and VAX004 vaccinees tested, but this activity could not be maintained. Similarly, ADCC was detected at mid-time points and declined almost to background levels after the last immunization. Neutralization and ADCC correlated with the strength of Abs to gp120, V1V2, and V3.

In contrast to neutralization and ADCC, which were detected in all individuals tested, V3-specific ADCP was induced in only a fraction of vaccinees. Moreover, its induction followed a distinct kinetics: ADCP was detected earlier and, although it declined, remained relatively high at the mid- and final time points. The V3-specific ADCP scores correlated with V3-binding Ab levels but not with V3 Ab-mediated neutralization. This observation suggests that ADCP is carried out by a distinct subset of V3 Abs from those mediating neutralization. All together the data demonstrate that Abs with diverse epitope specificity capable of mediating neutralizing and nonneutralizing activities were induced in VAX003 and VAX004 trial participants transiently at early to mid-time points of the immunization period.

In summary, functional V1V2- and V3-specific Abs were induced in VAX003 and VAX004 vaccine recipients, but these responses were not sustained in the late immunization period. Hence, research efforts are needed to develop Env-based immunogens that are more effective than native gp120 proteins in inducing potent and durable functional Abs to V1V2 and V3. This study also sheds light on the importance of monitoring vaccine-induced immune responses throughout the course of immunization.

## Electronic supplementary material

Supplementary Figure
