# Supplementary material for: Functional Antibody Response Against V1V2 and V3 of HIV gp120 in the VAX003 and VAX004 Vaccine Trials
Source: Sci Rep. 2018 Jan 11;8:542. doi: 10.1038/s41598-017-18863-0 (PMC5765017; doi:10.1038/s41598-017-18863-0)
Supplement: Supplementary file 1 — Supplementary Figure [file 41598_2017_18863_MOESM1_ESM.pdf]

Functional Antibody Response Against V1V2 and V3 of HIV gp120 in the VAX003 and VAX004 Vaccine Trials

Preetha Balasubramanian[1,2], Constance Williams[3], Mariya B. Shapiro[4,5], Faruk Sinangil[6], Keith Higgins[6], Arthur Nádas[7], Maxim Totrov[8], Xiang-Peng Kong[9], Andrew J. Fiore-Gartland[10], Nancy L. Haigwood[4,5], Susan Zolla-Pazner[2], Catarina E Hioe[2,11*]

[1]The Sackler Institute of Graduate Biomedical Sciences, NYU School of Medicine, New York, NY 10016, USA

[2]Department of Medicine, Division of Infectious Diseases, Icahn School of Medicine at Mount Sinai, New York, NY 10029, USA

[3]Department of Pathology, NYU School of Medicine, New York, NY 10016, USA

[4]Division of Pathobiology and Immunology, Oregon National Primate Research Center, Oregon Health & Science University, Beaverton, OR 97006, USA

[5]Department of Molecular Microbiology & Immunology, Oregon Health & Science University, Portland, OR 97239, USA

[6]Global Solutions for Infectious Diseases, South San Francisco, CA 94080, USA

[7]Department of Environment Medicine, NYU School of Medicine, New York, NY 10016, USA

[8]Molsoft LLC, La Jolla CA 92037, USA

[9]Department of Biochemistry and Molecular Pharmacology, NYU School of Medicine, New York, NY 10016, USA

[10]Vaccine and Infectious Disease Division, Fred Hutchinson Cancer Research Center, Seattle, WA, 98109, USA

[11]James J. Peters VA Medical Center, Bronx, NY 10468, USA

Word count: Abstract 199, Main Text 4057

*Corresponding author: Catarina E. Hioe, Department of Medicine – Division of Infectious Diseases, One Gustave L. Levy Place, Box 1090, New York, NY 10029 (catarina.hioe@mssm.edu).

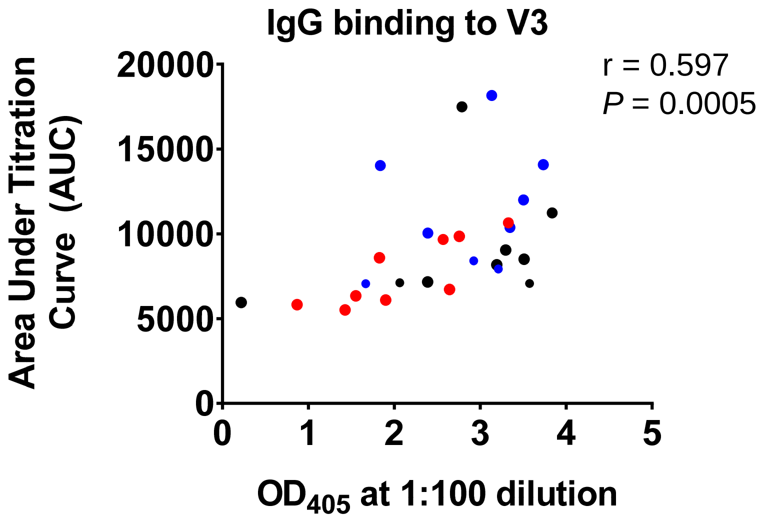

Supplemental Figure S1. Correlation between ELISA OD_{405} and AUC values for 6 VAX003 and 3 VAX004 vaccinees at early, mid-, and final time points against cyclic V3 MN. OD_{405} were determined with plasma samples diluted 1:100. AUCs were calculated from titration curves starting with 1:100, 3-fold down, for 8 dilutions. Correlation analysis was performed using the nonparametric Spearman test.
